# Detecting the Biopolymer Behavior of Graphene Nanoribbons in Aqueous Solution

**DOI:** 10.1038/srep31174

**Published:** 2016-08-09

**Authors:** Sithara S. Wijeratne, Evgeni S. Penev, Wei Lu, Jingqiang Li, Amanda L. Duque, Boris I. Yakobson, James M. Tour, Ching-Hwa Kiang

**Affiliations:** 1 Department of Physics and Astronomy, Rice University, Houston, TX 77005, USA; 2 Department of Material Science and NanoEngineering, Rice University, Houston, TX 77005, USA; 3 Department of Chemistry, Rice University, Houston, TX 77005, USA; 4 Richard E. Smalley Institute for Nanoscale Science & Technology, Rice University, Houston, TX 77005, USA; 5 Department of Bioengineering, Rice University, Houston, TX 77005, USA.

## Abstract

Graphene nanoribbons (GNR), can be prepared in bulk quantities for large-area applications by reducing the product from the lengthwise oxidative unzipping of multiwalled carbon nanotubes (MWNT). Recently, the biomaterials application of GNR has been explored, for example, in the pore to be used for DNA sequencing. Therefore, understanding the polymer behavior of GNR in solution is essential in predicting GNR interaction with biomaterials. Here, we report experimental studies of the solution-based mechanical properties of GNR and their parent products, graphene oxide nanoribbons (GONR). We used atomic force microscopy (AFM) to study their mechanical properties in solution and showed that GNR and GONR have similar force-extension behavior as in biopolymers such as proteins and DNA. The rigidity increases with reducing chemical functionalities. The similarities in rigidity and tunability between nanoribbons and biomolecules might enable the design and fabrication of GNR-biomimetic interfaces.

Nanoscale materials for biological application must be able to function properly in solution and interact favorably with biological nanomaterials. Graphene and related structures are emerging as potential biomaterials for a variety of applications^1^, so how their properties change in a biological environment will be crucial for the design and optimization of these materials with desired functionalities^2^,^3^,^4^,^5^. Among graphene-based materials, graphene nanoribbons (GNR) have attracted significant attention due to their high aspect ratio, unique electronic properties, mechanical strength, thermal conductivity and biocompatibility^6^. The long length of the GNR, here ranges from 1 to 5 μm, could render them a suitable biomaterial for *in vitro* biomedical applications^1^.

Among the known strategies for synthesizing GNR, the lengthwise oxidative cutting of multiwalled carbon nanotubes (MWNT) to generate graphene oxide nanoribbons (GONR), an oxide derivative of GNR, is a versatile method for large area applications^2^ (Fig. 1). During the oxidation, the π-bonds in the graphene network are disrupted, yielding oxygenated nanoribbon sheets covered with carbonyls, carboxyls and hydroxyls groups, resulting in highly water soluble structures of widths from 10 nm to more than 100 nm and lengths from 1 to 5 μm^7^,^8^. However, under hydrazine reduction, this network is reduced to form GNR.

Here we characterize the mechanical properties of GNR and their parent products, GONR, in aqueous solution by using AFM and have observed a unique mechanical behavior of these materials. Due to the one-dimensional like conformation of nanoribbons, we used experimental and analysis techniques similar to those used for the one-dimensional biological polymers. These experimental results suggested that signatures in the force curves may be affected by the wrinkles, loops, spirals, or other deformations in GNR. Understanding the mechanical properties of the nanoribbons in solution will aid in the design and fabrication of GNR-biomimetic interfaces.

## Results

Figure 2 shows the AFM pulling scheme of a single GNR. The AFM tip was brought into contact with the gold substrate and random segments of the GNR were stretched in aqueous solution at a pulling rate of 1 μm/s. Figure 3 shows the resulting force-extension curves for GONR and GNR. Only force-extension profiles displaying consistent features with a single detachment peak were considered for further analysis. Some of the force curves have a single force peak while others have multiple force drops and peaks, similar to those observed in forced unfolding of proteins or unzipping of nucleic acids. We separated force-extension curves into two groups: i) ones with single detachment peaks and ii) ones with multiple force peaks. A typical AFM image (Fig. 4) of GNR shows the morphology of these nanoribbons, which resembles the filamentous biopolymers such as proteins and DNAs. One hypothesis of the multiple force peaks is that these are from pulling a GNR polymer with wrinkles and loops. Because the non-linear behavior in the force-extension curves appears to be similar to that of biological molecules^9^,^10^,^11^, and our GNR/GONR are nearly one-dimensional, we first attempt to fit the force curves to the extensible wormlike chain (eWLC) model^12^,^13^ to obtain an estimate of the scale of bending and stretching energies,


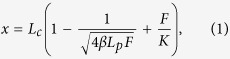


where *x* is the extension, *F* is the force, *K* is the elastic stretch modulus, *β* = 1/*k*_*B*_*T* where *k*_*B*_ is the Boltzmann constant, *T* is the temperature, *L*_*c*_ is the contour length and *L*_*p*_ is the persistence length.

Figure 5 shows the histogram distributions of the measured values of *L*_*p*_ and *K*. For GONR with only one force peak, the most probable values, resulting from Gaussian fits to histograms, are *L*_*p*_ = 15 nm and *K* = 3 nN (Fig. 5A,B). For GONR with multiple force peaks, the value of the peak force *F* = 170 pN (Fig. 5C). For GNR with only one force peak, the *L*_*p*_ = 35 nm and *K* = 1 nN (Fig. 5D,E). For GNR with multiple force peaks, the value of the peak force *F* = 120 pN (Fig. 5F). The measured *L*_*p*_ values agree well with reported values of 10–100 nm for GNR^14^,^15^. The *L*_*c*_ of 100–1500 nm for GNR are also consistent with reported values^2^.

## Discussion

The persistence length (*L*_*p*_) of GNR, obtained by fitting to the eWLC model, is comparable to that in other carbon materials, such as carbyne (one-dimensional chain of carbon atoms) or pristine GNR (Table 1). However, the stretch modulus (*K*) of GNR is 2–3 orders of magnitude smaller than the simulation results of carbyne and pristine GNR. Our experiments were carried out in aqueous solution, which may have an effect on their flexibility. In solution, GNR act more like a biopolymer, and thus the mechanical properties of GNR might depend on its environment. This has been demonstrated in cellulose paper, where a wet sheet had a lower strength and stiffness than a dry sheet^16^.

Loops and wrinkles in GNR are common^17^,^18^,^19^, and their mechanical properties can be probed by AFM force measurements. The existence of spirals, helicoids, wrinkles, and loops in GNR may explain the multiple kinks and force drops in our AFM experiments^15^. Force-drops have been observed in molecular simulations of GNR, as well as in functionalized GNR upon the application of a tensile force^20^.

The *K* = 1 nN and *L*_*p*_ = 35 nm of GNR are consistent with the values seen in double-stranded DNA (*ds*DNA). This may indicate that GNR and *ds*DNA are biocompatible mechanically and that GNR may form a structure or conformation similar to that of *ds*DNA (like a double helix)^20^. Both *ds*DNA and proteins can exist in different conformations, and GNR may contain different deformations and transform among those conformations when subject to external mechanical forces.

Because of the finite width of the graphene nanoribbons, GNR and GONR differ from the true one-dimensional biopolymers such as proteins and DNA, which can only form structures such as folds and loops. There have been a number of theoretical studies and models on the mechanical properties of ribbons, which can form a variety of structures such as helicoids, wrinkles, and spirals in additional to folds and loops. The ground state of nanoribbons can be helix or helicoid, depending on the width to thickness ratio of the material^21^,^22^. The chiral ribbon can form different structures such as helicoid and spiral, depending on a critical value, Föppl-von Kármán (FvK) number, which is determined by the competition between the bending and stretching energy^23^. When subjected to tension, the elastic macroscopic ribbons can undergo transitions between these morphologies^24^, which may be the origin of the kinks observed in the force curves. Furthermore, thermal fluctuations may have a nontrivial effect on the mechanics and give rise to *anomalous elasticity*, and its effect has been calculated to result in an enhanced bending rigidity and a suppressed stretching rigidity^25^. The increased bending stiffness with size in a two-dimensional graphene has been observed experimentally^26^ and the decrease in stretching stiffness may explain the lower than expected values of the stiffness *K* observed in our experiments of this nearly one-dimensional system. The simple eWLC model used in the current work does not take into account of these deformations, which may underlie the kinks and force drops in the observed force-extension curves that resemble biopolymer force curves. More theoretical and experimental studies are necessary to answer the questions about what effects the finite width of a nanoribbon, which constitutes the basic difference between the elasticity of filamentous and ribbon-like materials, have on the mechanics of nanoribbons.

The mechanical properties reported here provide an insight into the behavior of GNR in solution. Force-extension curves of GNR in aqueous solution possess similar features compared to those from biopolymers, such as proteins and DNA. GNR can have different phases that result in force drops when stretched with external forces. With these different morphologies, diverse biomimetic designed structures may be achieved by using GNR, which have potential applications in biomimetics with tunable properties.

## Methods

**Figure 1:**
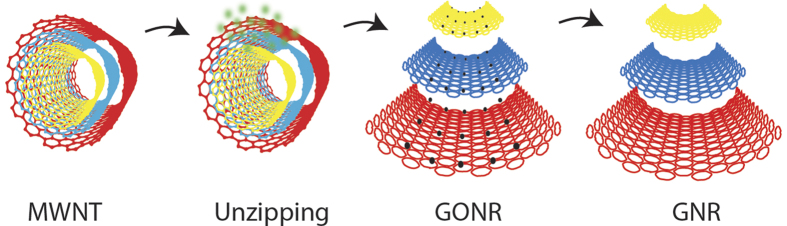
Illustration of graphene nanoribbon formation. The lengthwise cutting of MWNT to form GONR and GNR via oxidative shortening^2^. Chemical oxidants were used to unzip carbon nanotubes longitudinally to produce GONR. GONR are then reduced to form GNR.

GONR were made by oxidative unzipping of MWNT through permanganate oxidation^2^. The widths of the nanoribbons range from 10 nm to greater than 100 nm and lengths range from 1 to 5 μm after the reaction. These oxide samples were then reduced with hydrazine monohydrate to produce GNR (Fig. 1).

**Figure 2:**
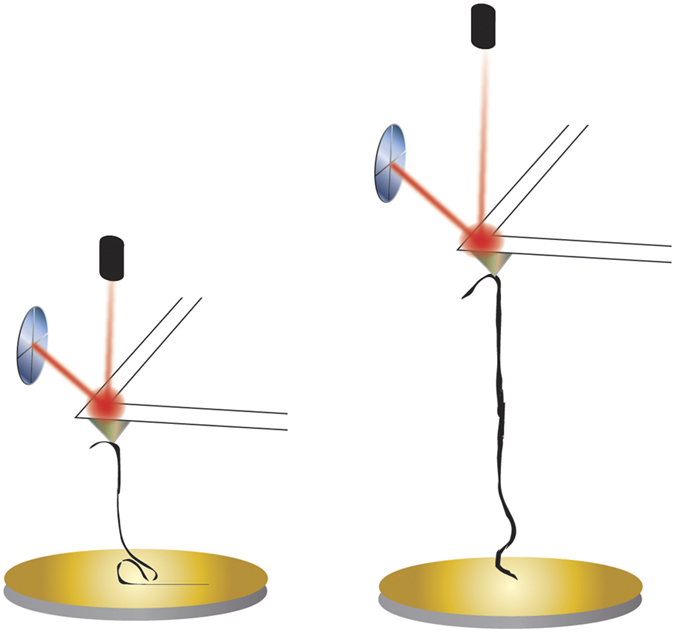
Illustration of graphene nanoribbon stretching. A schematic illustration of the AFM experiments on pulling a single nanoribbon. One end of GNR or GONR is attached to the gold substrate and the other end to the AFM cantilever. The forces on a single GNR or GONR were determined by the bending of cantilever.

**Figure 3:**
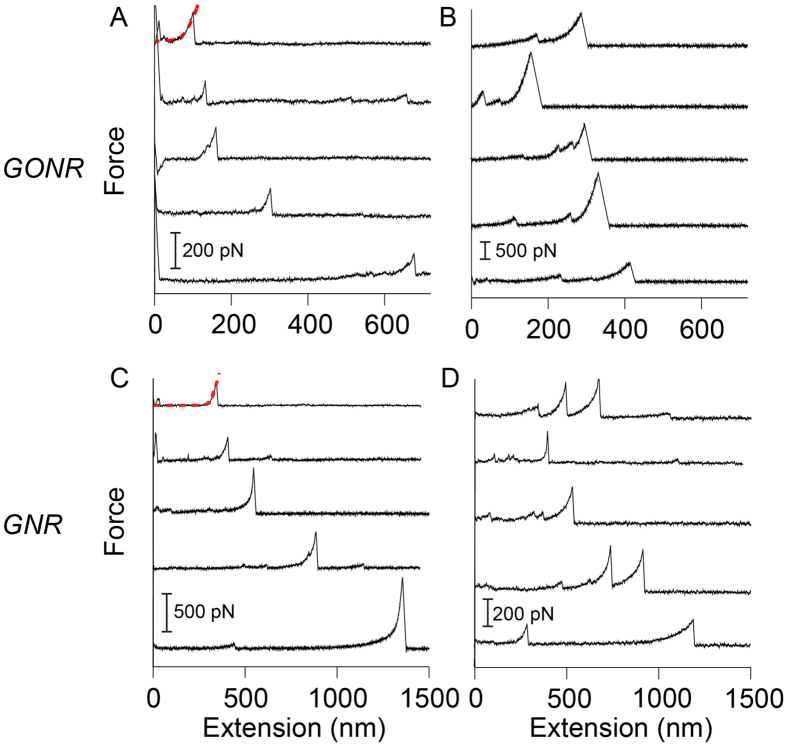
Force-extension curves of GNR and GONR. Force-extension curves of GONR show (**A**) a single force peak, and (**B**) multiple force peaks and kinks. Force-extension curves of GNR show (**C**) a single force peak, and (**D**) multiple force peaks and kinks. Force curves in (**A,B**) are from the same GONR sample, and (**C,D**) are from the same GNR sample. The dashed red curves are fits to the eWLC model, where *L*_*p*_ and *K* are determined (see Eq. 1).

**Figure 4:**
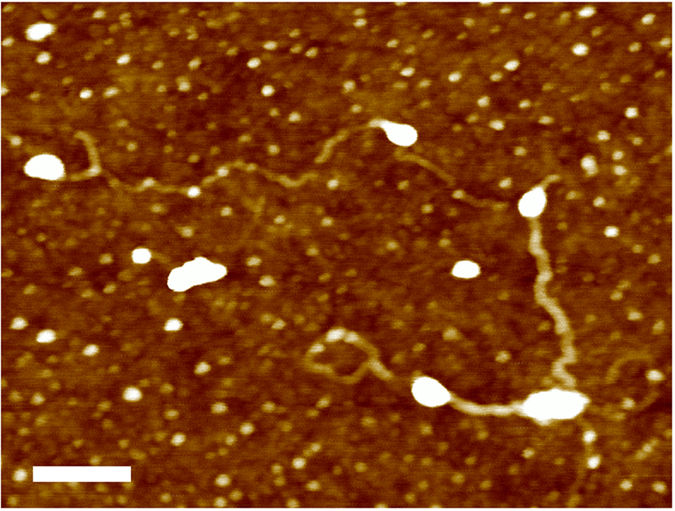
AFM image of graphene nanoribbons. AFM image of a typical graphene nanoribbon (GNR) derived from lengthwise unzipping of MWNT. The length is 1 μm and the width is 20 nm. These narrow GNR often have different morphologies, which include loops and often appear to be curved. Scale bar: 100 nm.

**Figure 5:**
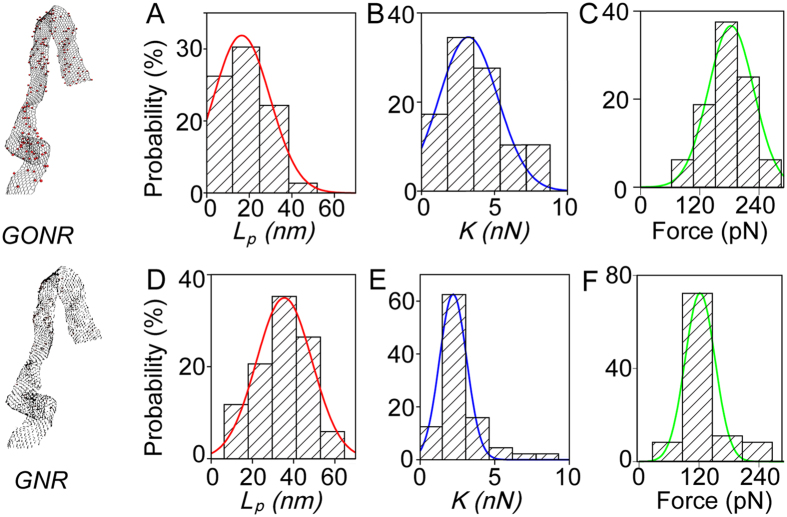
Experimental determination of mechanical parameters using one-dimensional polymer model. Data were fit with the eWLC model (Eq. 1) to determine *L*_*p*_ and *K*. For GONR curves with single force peaks, histograms show that (**A**) *L*_*p*_ = 15 nm and (**B**) *K* = 3 nN from fitting the distribution to a Gaussian curve. (**C**) For GONR with multiple force peaks the value of the peak force *F* = 170 pN. For GNR curves with single force peaks, histograms show that (**D**) *L*_*p*_ = 35 nm and (**E**) *K* = 1 nN. (**F**) For GNR curves with multiple force peaks the value of the peak force *F* = 120 pN.

Sample substrates for AFM experiments were prepared by allowing 10 μl of 0.1 mg/ml of the nanoribbon samples to absorb on to a fresh gold substrate for 10 minutes at room temperature. The substrate was then spincoated for one minute at 3,000 rpm to evenly distribute the nanoribbons on the surface. An AFM (Bruker, Inc.) was used to perform force-pulling measurements on the sample as illustrated in Fig. 2. Silicon nitride cantilevers with a spring constant of 0.04 N/m were used (MLCT, Bruker, Inc.). The nanoribbon samples were pulled in phosphate buffered saline (PBS, pH 7.4) at a pulling velocity of 1 μm/s. The force-extension curves obtained in each experiment were analyzed with a program written in MATLAB (MathWorks, Inc.). The data were binned into histograms and fit to a Gaussian curve. The error in the measurements is half of the bin width of the histograms.

To prepare the substrate for imaging, mica discs were glued to steel discs using epoxy and allowed to dry overnight. The dried mica disks were cleaved with scotch tape to reveal a fresh surface. For nanoribbon samples (0.1 mg/ml) of approximately, 10 μL were spincoated for one minute at 3,000 rpm, washed with Millipore water and then immediately dried with nitrogen gas and imaged in air. The ScanAsyst mode in air, using ScanAsyst air tips with a nominal spring constant of 0.4 N/m, was used for imaging.

## Additional Information

**How to cite this article**: Wijeratne, S. S. *et al*. Detecting the Biopolymer Behavior of Graphene Nanoribbons in Aqueous Solution. *Sci. Rep*. **6**, 31174; doi: 10.1038/srep31174 (2016).

## Figures and Tables

**Table 1:** Elastic properties of biopolymers and carbon materials.

Sample	*L*_*p*_ (nm)	*K* (nN)
*Biopolymers*		
Single-stranded DNA^27^	1–4	2
Native titin (folded)^28^	9–16	0.6–0.9
Native titin (unfolded)^29^	0.4	
Double-stranded DNA^13^	50	1
*Carbon materials*		
Carbyne*^30^	14	580
Pristine GNR*^14^	10–100	7800
GONR	15	3
GNR	35	1
SWNT^10^	32,000	45,000

GNR and GONR have bending and stretching rigidity on the same order of filamentous biopolymers such as proteins and DNA and much smaller than similar nanomaterials such as SWNT and two-dimensional graphene sheets.

^*^Simulation results.
